# Performance of subjective global nutritional assessment in predicting clinical outcomes: Data from the Brazilian survey of pediatric oncology nutrition

**DOI:** 10.1002/cam4.4837

**Published:** 2022-05-30

**Authors:** Wanélia Vieira Afonso, Wilza Arantes Ferreira Peres, Nivaldo Barroso de Pinho, Arthur Orlando Corrêa Schilithz, Renata Brum Martucci, Viviane Dias Rodrigues, Barbara Folino Nascimento, Carolina Ferraz Figueiredo Moreira, Patricia de Carvalho Padilha

**Affiliations:** ^1^ National Cancer Institute José Alencar Gomes da Silva (Instituto Nacional de Câncer José Alencar Gomes da Silva), Brazilian Ministry of Health (Ministério da Saúde do Brasil) Rio de Janeiro Brazil; ^2^ Josué de Castro Nutrition Institute (Instituto de Nutrição Josué de Castro) Federal University of Rio de Janeiro (Universidade Federal do Rio de Janeiro) Rio de Janeiro Brazil; ^3^ Brazilian Society of Oncological Nutrition (Sociedade Brasileira de Nutrição Oncológica) Rio de Janeiro Brazil; ^4^ Nutrition Institute (Instituto de Nutrição), State University of Rio de Janeiro (Universidade do Estado do Rio de Janeiro) Rio de Janeiro Brazil; ^5^ Institute of Childcare and Pediatrics Martagão Gesteira (Instituto de Puericultura e Pediatria Martagão Gesteira) Federal University of Rio de Janeiro (Universidade Federal do Rio de Janeiro) Rio de Janeiro Brazil; ^6^ Josué de Castro Nutrition Institute (Instituto de Nutrição Josué de Castro) Institute of Childcare and Pediatrics Martagão Gesteira (Instituto de Puericultura e Pediatria Martagão Gesteira), Federal University of Rio de Janeiro (Universidade Federal do Rio de Janeiro) Rio de Janeiro Brazil

## Abstract

**Background:**

Methods for assessing nutritional status in children and adolescents with cancer is a difficult in clinical practice. The study aimed to evaluate the performance of Subjective Global Nutritional Assessment (SGNA) in predicting clinical outcomes in children and adolescents with cancer in Brazil.

**Methods:**

This was a prospective cohort multicenter study. It was included 723 children and adolescents with cancer aged 2–18 years between March 2018 and August 2019. Nutritional assessment was performed according to World Health Organization recommendations and using SGNA within 48h of hospitalization. Unplanned readmission, length of hospital stay, and post‐discharge death were analyzed. Cohen’s kappa coefficient was used to ascertain the agreement between body mass index for age (BMI/A) and SGNA. The sensitivity, specificity, positive and negative predictive values, and accuracy of SGNA were estimated. Odds ratios (ORs) with 95% confidence intervals (CIs) were evaluated using multiple logistic regression.

**Results:**

The mean patient age was 9.4 ± 4.9 years. SGNA showed that 29.7% (*n* = 215) and 6.5% (*n* = 47) patients had moderate and severe malnutrition, respectively. Considering the concurrent validity criterion, SGNA had an OR (95% CI) of 6.8 (3.1–14.9) for predicting low and very low weight for age at admission, with a sensitivity and specificity of 72.4% (59%–82.1%) and 72% (64.2%–78.9%), respectively. SGNA could predict death in children with severe/moderate malnutrition, with an accuracy of 63.8% (63%–65.1%). Logistic multivariate analysis showed that the adjusted effect of death; hematological tumor; living in the northeast, southeast, and midwest regions of Brazil; and older age was associated with malnutrition according to SGNA.

**Conclusion:**

Based on concurrent validity between SGNA and anthropometry, SGNA performed well and had a good ability to predict death in Brazilian children with cancer.

## Abstract 2

This study used data from 723 children from the Brazilian Survey of Pediatric Oncology Nutrition and reported that more patients were classified as having malnutrition with SGNA than with classification of nutritional status based on body mass index.

**RESEARCH SNAPSHOT**

**Research question**

Does Subjective Global Nutritional Assessment (SGNA) perform well in assessing nutritional status and predicting clinical outcomes in children with cancer?

**Key findings**

This study used data from 723 children from the Brazilian Survey of Pediatric Oncology Nutrition and reported that more patients were classified as having malnutrition with SGNA than with classification of nutritional status based on body mass index.

## 1 INTRODUCTION

The quest for viable, efficient, and more comprehensive methods for assessing nutritional status in children and adolescents with cancer is a major challenge in clinical practice. Among several particularities of cancer, conventional methods used in the nutritional assessment of healthy children, especially those using body weight, have limitations when applied to children with cancer. More than 10% of these children's bodyweight can be influenced by their tumors, leading to underestimation or overestimation of the severity of malnutrition.^1^, ^2^ Although body mass index for age (BMI/A) is widely used in large population‐based surveys and preferred for its ease of execution in clinical practice, it is not an adequate option for children with cancer because of large tumor masses, organomegaly, amputation and edema, which often lead to erroneous nutritional diagnoses.^1^, ^2^, ^3^

Internationally, Subjective Global Nutritional Assessment (SGNA) is the most widely recommended tool for use in children and is recognized as the gold standard for the subjective assessment of nutritional status.^4^ SGNA was developed in Canada for use in children admitted to surgical units,^5^ with the original validation study demonstrating that moderate/severe malnutrition was associated with clinical outcomes, such as length of hospital stay and unplanned readmission.^6^

SGNA is a more robust instrument than anthropometry alone as it includes seven domains for assessing nutritional status (anthropometry, weight loss, dietary intake and habits, gastrointestinal patterns, functional capacity, and physical examination focused on nutrition) and is capable of covering different degrees of clinical complexity and comorbidities in the evaluation.^5^ SGNA has been tested in a population of children in intensive care^7^ and validated in Brazil for pediatric patients with acute disease.^8^It has been widely adopted as the gold standard in validation studies of nutritional screening instruments.^4^, ^9^

In Brazil, SGNA for patients with cancer has been validated for its semantic equivalence, translation, cross‐cultural adaptation, and content.^10^, ^11^ However, its predictive validity for real nutritional status needs to be assessed for oncological treatment in pediatric patients. Thus, this study aimed to assess the performance of SGNA in predicting clinical outcomes among children and adolescents hospitalized with cancer.

## 2 METHODS

### 2.1 Study design

This prospective cohort multicenter study is part of the Brazilian Survey of Oncology Nutrition in Pediatrics (IBNOPe) conducted at 13 specialized cancer units in Brazil. The study was conducted by the Brazilian Society of Oncology Nutrition in partnership with the National Cancer Institute (INCA) and Josué de Castro Nutrition Institute of the Federal University of Rio de Janeiro. The IBNOPe aimed to assess the prevalence of inadequate nutritional status in children and adolescents with cancer at the time of hospital admission in all regions of Brazil between March 2018 and August 2019.

### 2.2 Eligibility criteria

Patients of both sexes aged 2–18 years with confirmed malignancy who were undergoing treatment were eligible to participate in the study. Patients receiving palliative care (all patients with a cancer that was considered “not curable”) or intensive care; those with gene syndrome (like Down Syndrome and Beckwith Wiedemann), malformations, other chronic diseases such as kidney or heart disease; and those who were human immunodeficiency virus carriers were excluded.

### 2.3 Sample design and selection

The sample design was based on the assumption that the proportion of inadequate nutritional status in children diagnosed with cancer may vary across geographic regions.

The reference centers were selected from the National Information System on Health Establishments in a survey conducted in 2016, which had 44 hospitals registered in the country with 300 or more pediatric cancer hospitalizations per year, representing 97.3% of all pediatric oncology admissions in Brazil. The sample size in each stratum was calculated based on the rate of adequacy of nutritional status of children and adolescents in Brazil in 2016 (69%), with 5% significance, according to data from the Household Budget Survey.^12^ Due to the cluster sampling design, a design effect of 1.3 was used. In addition, the sample size had 80% power to detect differences in nutritional status between solid and hematological tumors in the order of 59% to 79% (differences of at least 20%) between large geographic regions. As the North region did not have the minimum number per stratum, it was used only to compose the estimate of the national proportion.

Initially, 15 hospitals and 1380 children and adolescents were sampled, corresponding to 34.9% of all hospitals with ≥300 hospitalizations in 2016. Proportional allocation was used to calculate the number of hospitalizations to be sampled per stratum, which was defined as at least 119 children (minimum number required for an inter‐regional comparison). This minimum number was not reached in the north region due to the overall lower number of hospitalizations. Due to the absence of data on unplanned readmission in the literature, the maximum data collection time at each institution was set at 1 year, between March 2018 and August 2019. With this time limit, the initial sample number was not reached because the number of unplanned readmissions was underestimated.

Once all data were collected, a calibration and sample expansion process was performed, which considered the initial weighting calculations (based on the sample characteristics), nonresponse rate, and number of first hospitalizations listed in the INCA Cancer Hospital Records, which indicated that 3600 children across the country were hospitalized for the first time for cancer care in 1 year. The list of first hospitalizations during the survey period was only published in the INCA Cancer Hospital Records after completion of the survey. These data were then used for weighting calculations with greater precision.

### 2.4 Anthropometry

Weight and height measurements were performed at hospital admission using standard practices recommended for the nutritional assessment of children and adolescents by the Ministry of Health^12^, ^13^ and the World Health Organization (WHO).^14^, ^15^ The following anthropometric indices were used: weight for age (W/A), height for age (H/A), and BMI/A. To assess concurrent validity, nutritional status was classified according to *z*‐scores using *z*‐score < −2 standard deviation (SD) as the cutoff point for any degree of malnutrition (low and very low weight for age, thinness, marked thinness, and short and very short stature for age) as recommended by the WHO. Anthro and AnthroPlus software (both, version 3.2.2) were used for children aged ≤ 5 years and those aged > 5 years and adolescents, respectively.^16^

*Z*‐score at <−1 to indicate below‐adequate BMI/A (underweight or risk of underweight), signifying children at nutritional risk, for the purpose of comparison as cancer is associated with at potentially high nutritional risk in this population.^17^

### 2.5 SGNA in pediatrics

The SGNA questionnaire has been translated to Brazilian Portuguese and cross‐culturally adapted for use in Brazilian children and adolescents aged 2–18 with cancer.^11^ SGNA was administered to all study participants up to 48 h after hospitalization. In the first part of the questionnaire, the clinical history of the patients was assessed with a nutritional focus. It involved questions about adequacy of height and current weight, unintentional weight changes, adequacy of dietary intake, gastrointestinal symptoms (signs and frequency), functional capacity, and metabolic stress (physiological and metabolic changes caused by the clinical condition) and physical examination to assess for loss of subcutaneous fat in the regions of the cheeks, biceps, triceps, and ribs; muscle loss in the region of the clavicle, shoulder, scapula, quadriceps, knee, and calf; and presence of edema (ankle and sacrum).

The second part of the questionnaire involved dietary recall, with questions on food consumption, frequency of food intake, eating habits, and physical and functional activities. Nutritional status was classified as normal or well‐nourished, moderately malnourished, or severely malnourished, according to the guidelines for the use of SGNA in pediatrics.^5^, ^11^

### 2.6 Data quality

In each participating institution, a dietitian with experience in pediatrics was the designated supervisor. Every time data were collected, the forms were immediately reviewed by the team and the field supervisor to minimize potential data‐filling errors. Data entered in the online form were reviewed by two different supervisors at each institution and later by the main researcher and research team to identify any inaccuracies.

To answer queries related to data collection, the members of the executive research team were on call 7 days a week on a rotational basis throughout the collection period and via a digital platform.

The computerized system was developed using Visual Studio (Microsoft) and the C‐SHARP programming language. MySql was used as the database. The program was developed to work on the Internet. The information was entered by each reference center participating in the study. All existing information was related to a specific patient for a specific institution previously registered in the system by the supervisor at each reference center.

### 2.7 Clinical outcomes of interest and clinical information

The following outcomes were analyzed to assess the performance of SGNA: unplanned readmission, defined as unplanned readmission because of clinical complications within 30 days of discharge (yes/no); length of hospital stay >7 days (yes/no); and death within 60 days (yes/no).

Disease duration was defined as the time from diagnosis to the date of data collection. Treatment time defined as the time elapsed between the start of treatment and data collection.

### 2.8 Ethical aspects of research

This study approved by the research ethics committee (CAAE 72541617.8.1001.5274) of all participating reference centers, as required in Resolution 466/2012 concerning research involving humans.^18^ For children aged <12 years, their guardians provide informed consent, while all adolescents aged ≥12 themselves provided informed assent.

### 2.9 Data quality

Practical training was provided on how to conduct the nutritional assessment, how to administer the SGNA, and how to input the data in the platform. The records and collection forms were reviewed by the field supervisor, and the information entered in the online form was checked by two different supervisors at the institution. All data were checked by the main researcher and research team in order to identify any inaccuracies.

### 2.10 Data analysis

Normality tests were performed using the Shapiro–Wilk test, graphic analyses, and the coefficients of asymmetry and kurtosis. Continuous variables with normal distribution were analyzed using Student's *t*‐test, while the variables with nonnormal distribution were compared across the nutritional risk categories using the Kolmogorov–Smirnov test. The association between categorical variables and nutritional risk was verified using Pearson's chi‐squared test or Fisher's exact test. Agreement between the classifications according to BMI/A and SGNA was assessed using Cohen's kappa coefficient; kappa values of 0–0.19, 0.20–0.39, 0.40–0.59, 0.60–0.79, and 0.81–1.00 indicated very poor, poor, moderate, good, and excellent agreement, respectively.^19^

Concurrent criterion validity was assessed based on the sensitivity, specificity, positive predictive value (PPV), negative predictive value (NPV), and accuracy of SGNA for the detection of acute (W/A), chronic (H/A), and general (BMI/A) malnutrition.

Predictive validity was assessed using the same indices used to identify length of hospital stay (>7 days), unplanned readmission within 30 days of hospital discharge, and death within 60 days of hospital discharge.

To assess the association between outcomes and factors according to the occurrence of malnutrition, univariate logistic analysis was performed to estimate the odds ratio (OR) with 95% confidence interval (CI; significance level of 5%). Multivariate analyses were performed using a manual stepwise method to evaluate possible associations adjusted for factors with a *p*‐value of <0.250 in univariate analysis (Hosmer and Lemeshow, 2000). After testing insertion one by one, only those that remained significant were analyzed (*p* ≤ 10%). SPSS Statistics for Windows, version 26,^20^ was used for statistical analyses.

## 3 RESULTS

**FIGURE 1:**
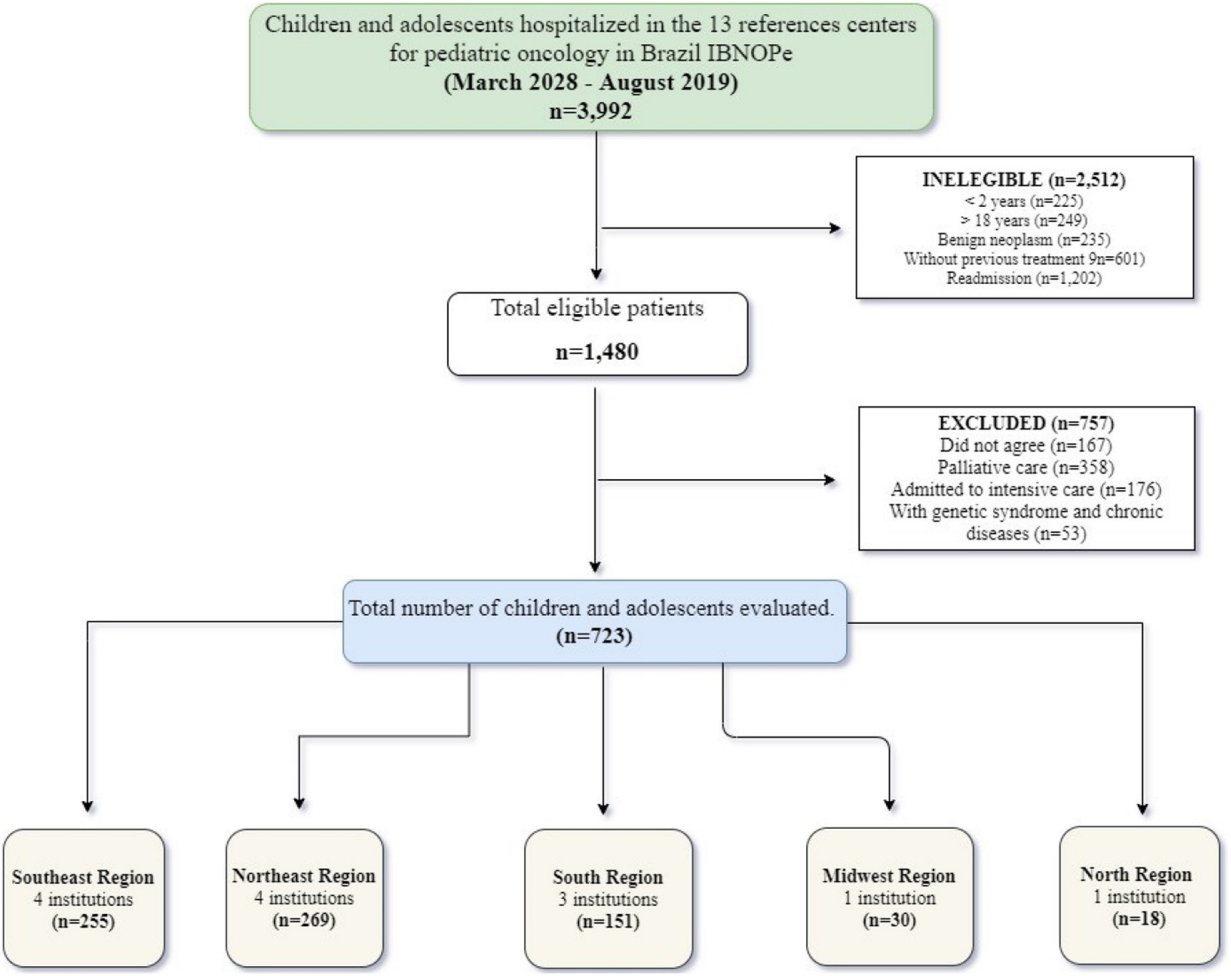
Flowchart of the sample of the Brazilian Survey of Oncological Nutrition in Pediatrics. IBNOPe. Brazil.

The sample included 723 children and adolescents (Figure 1) with a mean age of 9.38 ± 4.88 years. Leukemia and lymphoma were the most prevalent types of cancer (62.2%, *n* = 450). According to the anthropometric index, 10.7% (*n* = 78) patients were markedly thin or thin, 64.3% (*n* = 465) patients had normal weight, and 25% (*n* = 181) patients were overweight or obese. Regarding height, 6.1% (*n* = 4) patients were classified as being short or very short for age, with most patients also having poor nutritional status. According to SGNA, 29.7% (*n* = 215) patients had moderate malnutrition and 6.5% (*n* = 47) patients had severe malnutrition.

Table 1 shows the characteristics of the patients according to the SGNA classification, with moderate/severe malnutrition being more prevalent among adolescents aged 10–18 years (*p* = 0.015), patients with solid tumors (*p* = 0.014), patients with longer disease duration (*p* = 0.014), and patients who were categorized as being thin according to BMI/A (*p* < 0.001).

**TABLE 1:** Characteristics of children and adolescents in the Brazilian Survey of Pediatric Oncology Nutrition study sample according to the SGNA classification for children with cancer (*n* = 723)

Variables	SGNA classification for children				
	Well‐nourished		Moderately/severely Malnourished		*p*‐value
	*n* (%)	IQR	*n* (%)	IQR	
Sex (*n* = 723)					
Male	272 (61.3)		172 (38.7)		0.104
Female	189 (67.7)		90 (32.3)		
Age (in years) (*n* = 723) (median and IQR)	8.8		10.3	(6.3–14.1)	<0.001
Tumor type classification (*n* = 723)					
Solid tumors	151 (55.3)		122 (44.7)		0.014
Hematological	310 (68.9)		140 (31.1)		
Current treatment – Chemotherapy only (*n* = 723)					
Yes	409 (65.9)		212 (34.1)		0.146
No	52 (51.0)		50 (49.0)		
Weight measured at admission (kg) (median and IQR) (*n* = 723)	26.0	(17.8–47.0)	29.2	(19.0–43.7)	0.019
*Z*‐score for weight for age at admission (W/A) (mean and SD) (*n* = 723)	0.30 (1.12)		−0.63 (1.25)		<0.001
Height measured at admission (cm) (mean and SD) (*n* = 716)	129.1 (28.3)		136.5 (26.4)		0.001
*Z*‐score for height for age at admission (H/A) (mean and SD) (*n* = 716)	−0.14 (1.33)		−0.40 (1.18)		0.007
BMI at admission (mean and SD) (*n* = 716)	18.3 (4.0)		16.2 (3.2)		<0.001
*Z*‐score for BMI at admission (BMI/A^a^) (mean and SD) (*n* = 716)	0.49 (1.31)		−0.98 (1.61)		<0.001^b^
Time between cancer diagnosis and admission to hospital (in days) (median and IQR) (*n* = 721)	119.0	(47.6–264.0)	168.7	(54.3–432.7)	0.014^c^
Length of stay (LOS) (in days) (median and IQR) (*n* = 723)	7.0	(4.0–15.3)	6,0	(4.0–14.0)	0.729^d^

Abbreviations: BMI, body mass index; H/A, height for age; IQR, interquartile range; SD, standard deviation; SGNA, Subjective Global Nutritional Assessment; W/A, weight for age.

^a^ BMI/A, body mass index for age. Short and very short height = H/A ≥ –3 and < −2 z‐score and < −3 z‐score, respectively. Thinness and marked thinness = BMI/A ≥ –3 and < −2 z‐score and < −3 z‐score, respectively.

^b^ Chi‐square test.

^c^ Komogorov–Smirnov test.

^d^ Student's *t*‐test.

Table 2 shows a comparison of the SGNA and anthropometric classifications. The SGNA classification showed little agreement with the BMI/A classification. According to SGNA, 69.5% patients were categorized into the normal or well‐nourished group, while according to BMI/A, 83.5% patients were categorized into the moderately or severely malnourished group. Only 25.4% patients categorized into the moderately or severely malnourished group by SGNA were identified to be moderately or severely malnourished by BMI/A, demonstrating the weakness of the anthropometric indicator for classifying malnutrition in pediatric patients with cancer. When considering the nutritional risk value (*z*‐score = −1), the level of agreement was *k* = 0.265.

**TABLE 2:** Agreement between Subjective Global Nutritional Assessment (SGNA) and anthropometric assessment using body mass index for age (BMI/A) at the time of admission in the Brazilian Survey of Pediatric Oncology Nutrition (*n* = 723)

	SGNA				Kappa^a^
	Moderately/severely malnourished		Well‐nourished		
	*n*	% of SGNA category	*n*	% of SGNA category	
BMI/A					
Thinness/marked thinness (% of BMI/A category)	66	83.5	13	16.5	
		25.4		2.9	0.265
Normal weight/overweight/obesity (% of BMI/A category)	194	30.5	443	69.5	
		74.6		97.1	

*Note*: BMI/A categories: <−3 *z*‐score = marked thinness, ≥−3 and <−2 *z*‐score = thinness, ≥−2 and <+1 *z*‐score = normal weight, ≥+1 and <+2 *z*‐score = overweight, ≥+2 *z*‐score = obesity.

Abbreviations: BMI/A, body mass index for age; SGNA = Subjective Global Nutritional Assessment.

^a^ Cohen's kappa coefficient (significance at 5%).

The median length of hospital stay was 7 days. Further, 62% (*n* = 448) patients were readmitted to the hospital within 30 days of hospital discharge and 4.3% (*n* = 31) died within 60 days of hospital discharge.

The concurrent validity assessment demonstrated that the sensitivity and specificity of SGNA was 72.4% and 72.4%, respectively, for acute malnutrition (weight/age) and 83.5% and 69.5%, respectively, for marked thinness or thinness, defined according to the WHO cutoff point.^14^, ^15^

The ability of SGNA for accurate classification was 72.1% for acute malnutrition and thinness, defined according to the WHO classification, and 76.2% for nutritional risk, based on a *z*‐score of <−1 for BMI/A. Predictive validity assessment demonstrated that severe/moderate malnutrition according to SGNA was able to predict an appropriately two times higher risk of death among the study population (Table 3).

**TABLE 3:** Criterion validity (concurrent and predictive) of the Subjective Global Nutritional Assessment of the Brazilian Survey of Pediatric Oncology Nutrition sample (*n* = 723)

	OR (95% CI)	SE (95% CI)	SP (95% CI)	PPV (95% CI)	NPV (95% CI)	ACC (95% CI)
Criterion validity (concurrent)						
Low and very low weight for age at admission (W/A)^a^ (*n* = 29/401)	6.8 (3.1–14.9)*	72.4 (59.9–82.1)	72.0 (64.2–78.9)	16.8 (9.5–28.9)	97.1 (92.6–98.8)	72.1 (68.3–74.1)
Short and very short stature for age at admission (H/A)^b^ (*n* = 42/716)	0.8 (0.5–1.5)	3.3 (22.8–44.1)	63.5 (55.0–71.2)	5.4 (2.4–11.1)	93.9 (88.7–98.7)	61.7 (61.3–62.6)
Thinness and accentuated thinness on admission (BMI/A)^c^ (*n* = 79/716)	11.9 (4.9–24.6)*	83.5 (66.8–93.1)	69.5 (61.5–76.6)	25.4 (19.5–32.1)	97.1 (94.4–98.7)	71.1 (69.3–71.8)
Anthropometric assessment below adequate at admission (BMI/A^c^ *Z*‐score ≤ −1) (*n* = 172/723)	10.1 (6.1–16.8)*	76.2 (63.5–85.3)	76.2 (68.6–82.4)	50.0 (42.4–57.3)	91.1 (87.0–94.0)	76.2 (72.2–77.7)
Criterion validity (predictive)						
Length of hospital stay<7 days^d^ (*n* = 323/723)	0.9 (0.5–1.5)	35.0 (25.1–46.5)	62.8 (53.6–71.1)	43.1 (25.8–62.5)	54.4 (40.0–68.1)	50.3 (43.7–53.1)
Unplanned readmission (*n* = 439/723)	0.7 (0.5–1.0)	33.0 (26.0–40.7)	58.6 (48.5–68.1)	55.1 (43.2–66.5)	36.2 (27.6–45.7)	43.2 (36.3–46.0)
Death (*n* = 30/723)	1.7 (1.2–2.5)*	50.1 (37.5–60.4)	64.4 (56.1–71.7)	5.7 (3.6–8.8)	96.7 (94.4–98.0)	63.8 (63.0–65.1)

Abbreviations: BMI/A, body mass index for age; ACC, accuracy; CI, confidence interval; H/A, height for age; OR, odds ratio; NPV, negative predictive value; PPV, positive predictive value; SE, sensitivity; SP, specificity; W/A, weight for age.

^a^ Weight for age <−2 *z*‐score (<5 years).

^b^ Height for age <−2 *z*‐scores (all ages).

^c^ BMI for age <−2 *z*‐score (<5 years) or BMI for age <−2 z‐score (≥5 years).

^d^ Categorization according to median: ≤7 days; >7 days; *p < 0.05.

The factors associated with moderate/severe malnutrition according to SGNA in multivariate logistic regression were hematological tumors; living in the southeast, northeast, and midwest regions of Brazil; older age; time of diagnosis; and death (Table 4).

**TABLE 4:** Multiple logistic regression of patient factors associated with moderate/severe malnutrition according to SGNA in the Brazilian Survey of Pediatric Oncology Nutrition sample (*n* = 723)

	*p*‐value^a^	Odds ratio	95% CI	
			Lower	Upper
Sex (Male)				
Female	0.075	0.75	0.53	1.03
Region (South)				
North	0.493	0.59	0.16	1.49
Northeast	0.015	1.90	1.32	3.21
Midwest	0.013	2.91	1.25	6.78
Southeast	0.030	1.67	1.05	2.75
Tumor type classification (Solid)				
Hematological	<0.001	0.56	0.40	0.77
Age (years)	0.001	1.05	1.02	1.09
Time of diagnosis (months)	0.005	1.02	1.01	1.03
Death (yes)	0.069	2.11	0.95	4.72

^a^ Wald'stest.

## 4 DISCUSSION

Nutritional assessment showed a high prevalence of inadequate nutritional status according to the pediatric SGNA, with >30% patients having moderate/severe malnutrition. In the criterion validity assessments, both concurrent and for predicting death within 60 days of hospital discharge, SGNA demonstrated a satisfactory performance.

Considering the general characteristics of the sample, malnutrition, identified by SGNA, was more prevalent among patients with solid tumors, which is consistent with the literature on the nutritional status of children with cancer.^21^, ^22^ In a recent systematic review on the association between nutritional status and treatment‐related toxicity, disease‐free survival, the cumulative incidence of relapse, and overall survival in children and adolescents with solid tumors, approximately 62% patients were overnourished or undernourished (according to BMI/A) at the time of diagnosis. In a previous study, abnormal body mass index (BMI) was associated with worse overall survival in Ewing's sarcoma (relative risk [RR] = 3.46; *p* = 0.022) and osteosarcoma (RR = 1.6; *p* < 0.005), and a trend in worse overall survival was found in rhabdomyosarcoma (RR = 1.70; *p* = 0.0596).^23^

Considering the particularities of this disease, proper identification of the nutritional status of children hospitalized with cancer is a major challenge that requires a broader and more insightful perspective on nutritional assessment.^23^, ^24^ Methods based on subjective criteria have been investigated as viable alternatives, either on their own or to supplement classic methods such as anthropometry.^25^ However, most of the instruments available and some instruments validated in Brazil are inadequate for the diagnosis of nutritional status as they are screening instruments for tracking nutritional risk. However, none have been applied to children with cancer in Brazil.

In this cohort, the agreement between the diagnosis of malnutrition at admission by anthropometry and SGNA was considered weak. When SGNA was compared with BMI/A (WHO classification), it was found that most children and adolescents were classified as being well‐nourished and of normal weight. There was poor agreement with the cutoff points for marked thinness and thinness (*z*‐scores = −2 and −3; *k* = 0.265; Table 2) and nutritional risk (*z*‐score = −1; *k* = 0.440); the cutoff point for the latter is usually applied in the nutritional assessment of children and adolescents.

These data corroborate the findings of other recent studies in Brazil with other patient populations.^8^, ^26^ Carniel et al.^8^ observed poor agreement (*k* = 0.38; *p* = 0.001) between weight/height and SGNA, while Pimenta et al.^26^ found poor agreement (*k* = 0.21; *p* = 0.001) between SGNA and BMI/A.

Such differences may be explained by the fact that SGNA comprises qualitative questions. This makes it efficient in identifying risks and nutritional changes early,^24^ as evidenced by the greater agreement with nutritional risk, even if only slight (*z*‐score:<−1). It can be used to identify significant alterations in the nutritional status of children and adolescents that classic measures and indices of nutritional assessment, such as the exclusive use of the WHO classification,^27^, ^28^ cannot detect, especially in patients with chronic diseases.

Thus, SGNA is a more robust assessment than each anthropometric measure alone as it includes important domains of nutritional status and physical examination with a focus on nutrition. There are some instruments in the literature for nutritional screening in pediatric patients,^9^, ^28^, ^29^ with some studies interpreting SGNA as a nutritional screening method.^28^, ^30^, ^31^ However, SGNA is the only method used for subjective assessments, with most studies using it as the “gold standard” for assessing the performance of nutritional screening tests.^7^, ^11^

However, the methodology for validating subjective screening/assessment tools is unclear in the literature as there is no universally accepted gold standard for comparison. In practice, assessments of this kind involve the analysis of issues such as reproducibility, applicability, and validity regarding the prediction of malnutrition and other results.^32^ Higher sensitivity values than specificity values are desirable for screening as the latter is associated with failure to identify individuals at risk; in other words, it leads to a lower number of false‐negative results.^33^

The concurrent validity assessment showed good sensitivity, specificity, and accuracy, confirming the good performance of SGNA, as suggested for the evaluation of accuracy tests.^34^, ^35^ This result is consistent with that reported in studies using nutritional screening methods.^30^, ^36^ Carter et al.^9^ undertook a study to determine the best nutritional screening tool for identifying malnutrition in children at hospital admission. They assessed the concurrent validity of the Pediatric Nutrition Screening Tool (PNST) against SGNA and the WHO‐recommended cutoff points (*z*‐score ≤ −2) and found a sensitivity of 58%, specificity of 88%, PPV of 67%, and an NPV of 83% for PNST. A similar analysis was performed by Huysentruyt et al.^34^ regarding the choice of cutoff points owing to their great impact on the performance of tools. However, SGNA does not aim to use cutoff points as it values subjectivity and focuses primarily on nutritional semiology.

Another study with hospitalized children conducted in Malaysia^29^ found that the SGNA had concurrent and predictive validity and was able to classify a greater number of children with malnutrition (68%) than PNST (57%). SGNA had greater sensitivity (87.8%) and specificity (70.6%) and a lower rate of false negatives (12.2%). The PPV of SGNA indicated that 85.2% children who were identified as malnourished actually had malnutrition.^29^ SGNA is recognized as a more structured nutritional assessment tool rather than a nutritional risk tracking tool, which means it has consolidated nutritional diagnostic capacity.

In the validation of SGNA in Brazil for children hospitalized with different diseases, Carniel et al.^8^ found an approximately four times higher probability of hospitalization/unplanned readmission in patients with moderate malnutrition than in patients with normal weight. Among patients with severe malnutrition, the probability of hospitalization/unplanned readmission was five times higher (OR = 4.97; 95% CI = 2.61 9.48). However, no association was found between length of hospital stay and SGNA, corroborating our findings, but inconsistent with the findings of other studies,^6^, ^37^, ^38^ including those of the original validation study.^6^

Although an increasing number of studies have corroborated the relationship between nutritional status and mortality, especially in chronic diseases, few studies have considered this outcome using SGNA. The first study to address this relationship was the original SGNA validation study; however, all the findings showed that all deaths occurred due to infectious complications, regardless of nutritional status.^6^Studies based on anthropometric measurements have already shown the relationship between nutritional status and death,^23^ demonstrating the impact of malnutrition on the lethality of diseases. Our study is one of the few studies to establish such a relationship using SGNA.

MUAC has been recommended in the nutritional assessment of children with cancer, but it was decided not to use this method in the study because it does not clearly distinguish the affected body compartment, therefore, it would not add further analysis.

The addition of children with solid tumors did not affect the results. This analysis was included in the study design. However, because it is not possible to infer the influence of tumor size on BMI, the use of the SGNA instrument is proposed to solve this problem. Solid tumors were considered in multivariate analysis, revealing their influence independent of other factors on nutritional status.

The strengths of this study are that it is part of the largest Brazilian study on the nutritional status of hospitalized children with cancer, includes a representative sample of the Brazilian population, uses objective and subjective data for the nutritional assessment of this population, and is the first study, to our knowledge, to assess the steps for validating the use of SGNA in pediatric patients with cancer. Its limitations include the absence of data on the outcome of infections, need for specialized nutritional therapy for validation, and lack of food intake data. However, SGNA contains a broad, organized questionnaire with items on eating habits and food consumption to assess dietary adequacy; thus, this information is covered in the subjective nutritional assessment. The comparison of BMI alone with SGNA is also a limitation of this study, especially when patients with solid tumors were not excluded, but this analysis was included in the study design. However, because researcher could not infer the influence of tumor size on BMI, it was proposed the use of the SGNA instrument to solve this problem. Solid tumors were considered in the multivariate analysis, revealing their influence independent of other factors on nutritional status.

In terms of practicality, in a previous study of cross‐cultural adaptation, semantic evaluation, and content validation of SGNA, its completion time was found to be 15 min on average.^11^ Carter et al.^9^ cites <5 min for filling out the screening instruments and 15–30 min for SGNA. Given the broad interpretation of nutritional status according to SGNA and the complexity of cancer, this time is considered reasonable.

## 5 CONCLUSION

The results of this study indicated that SGNA could be used to complement classic nutritional assessment. Based on concurrent validity between SGNA and anthropometry, SGNA performed well and had a good capacity to predict death in Brazilian children with cancer. Children enrolled in the study with hematological cancers had a lower risk of malnutrition than those with solid tumors. Older age and living in the northeast, southeast, and midwest regions of Brazil were associated with a higher risk of malnutrition. The validation steps performed in this study showed that the SGNA performed well in assessing the nutritional status of Brazilian children and adolescents with cancer.

## CONFLICT OF INTEREST

The authors have no conflict of interest to declare.

## Data Availability

The data are not publicly available due to privacy or ethical restrictions.
